# The impact of rumen-protected amino acids on the expression of key- genes involved in the innate immunity of dairy sheep

**DOI:** 10.1371/journal.pone.0233192

**Published:** 2020-05-14

**Authors:** Eleni Tsiplakou, Alexandros Mavrommatis, Dimitrios Skliros, Federico Righi, Emmanouil Flemetakis

**Affiliations:** 1 Department of Animal Science, Laboratory of Nutritional Physiology and Feeding, School of Animal Biosciences, Agricultural University of Athens, Athens, Greece; 2 Department of Biotechnology, Laboratory of Molecular Biology, School of Food, Biotechnology and Development, Agricultural University of Athens, Athens, Greece; 3 Department of Veterinary Science, University of Parma, Parma, Italy; University of Illinois, UNITED STATES

## Abstract

Rumen protected amino acids inclusion in ewes’ diets has been proposed to enhance their innate immunity. The objective of this work was to determine the impact of dietary supplementation with rumen-protected methionine or lysine, as well as with a combination of these amino acids in two different ratios, on the expression of selected key-genes (NLRs, MyD88, TRIF, MAPK-1, IRF-3, JunD, TRAF-3, IRF-5, IL-1α, IL-10, IKK-α, STAT-3 and HO-1). Thus, sixty Chios dairy ewes (*Ovis aries*) were assigned to one of the following five dietary treatments (12 animals/ treatment): A: basal diet consist of concentrates, wheat straw and alfalfa hay (control group); B: basal diet **+**6.0 g/head rumen-protected methionine; C: basal diet + 5.0 g/head rumen-protected lysine; D: basal diet **+**6.0 g/head rumen-protected methionine + 5.0 g/head rumen-protected lysine and E: basal diet **+**12.0 g/head rumen-protected methionine + 5.0 g/head rumen-protected lysine. The results revealed a significant downregulation of relative transcript level of the IL-1α gene in the neutrophils of C and in monocytes of D ewes compared with the control. Significantly lower mRNA transcript accumulation was also observed for the MyD88 gene in the neutrophils of ewes fed with lysine only (C). The mRNA relative expression levels of JunD gene were highly induced in the monocytes, while those of IL-10 and HO-1 genes were declined in the neutrophils of ewes fed with the C and D diets compared with the control. Lower transcript levels of STAT-3 gene were observed in the neutrophils of ewes fed with either C or with E diets in comparison with the control. In conclusion, our results suggest that the dietary supplementation of ewes with rumen-protected amino acids, down regulate the expression of some genes involved in the pro-inflammatory signalling.

## Introduction

The intensive farming of high genetic merit ruminants can be associated with several issues including immunosuppression, which makes them susceptible to infections with negative consequences in their wellbeing and production [1]. A strengthen of animals’ immune system is close related with farm sustainability. However, the enhancement of animals’ immune system the last years can be achieved by alternative nutritional strategies than that of antibiotics, such as the use of amino acids etc.

Proper nutrition is considered as one of the most important factors in maintaining a functional immune system and preventing inflammation-inducing conditions such as tissue damage, metabolic syndrome-related disorders, etc. [2, 3]. To this purpose, during recent years, amino acids are gaining interest as a mean factor for the nutrition-mediated improvement of the immune system. Transcriptional studies have shown that genes related with inflammation in calves polymorphonuclear leukocytes are significantly downregulated, both *in vivo* [4] and *in vitro* [5], when the animals consumed methionine alone or in combination with choline and taurine respectively. Furthermore, a reduction in the proinflammatory status of dairy cows when the animals were fed with rumen protected methionine [4, 6, 7] has been observed, during the peri-parturient period.

However, inflammation can occur in high-producing small ruminants throughout their lactation period, since they are fed on a group basis and the amount of feed which they consumed is derived from the mean nutritional requirements of the entire flock, which drives usually to under and/or over- feeding incidences. Both under- and overfed animals have high non-esterified fatty acids (NEFA) and β-hydroxybutyric acid (BHBA) content in their blood, both of which affect the immune response [8]. Indeed, it has been found that chronically raises concentrations of NEFA in dairy cows, lead to reduced viability of polymorphonuclear neutrophil leukocytes *in vitro*, which could reduce the immune response to pathogens [9]. However, to the best of our knowledge, scarce information exists on the impact of rumen-protected amino acids on the expression of genes involved in the immune systems of high-producing sheep [10]. More specifically, the dietary supplementation with rumen-protected amino acids such as methionine and/or lysine not only downregulates the expression levels of proinflammatory cytokines (IL-1β, TNF-α), chemokine (CXCL-16) and pathogens recognition receptor-4 (TLR-4) but reduces significantly the BHBA content in ewes [10].

The immune system can be divided into the innate (nonspecific) immune system and the acquired (specific) immune system [11]. White blood cells (macrophages and neutrophils) belong to the innate immune system and are the “first responders” to pathogens and infections. Thus, both macrophages and neutrophils express several families of pattern-recognition receptors (PRRs) such as NOD-like receptors (NLRs) that recognise the different pathogen-associated, molecular pattern (PAMPs) [12]. Signal transduction by PRRs, including NLRs, depends on their conjunction with specific adaptors such as Myeloid-Differentiation-primary response gene 88 (MyD88) and TIR (Toll/Interleukin-1 Receptor) domain-containing adaptor protein inducing interferon beta (TRIF). The MyD88 signaling pathway regulates the transcription factor Mitogen-Activated Protein Kinase-1 (MAPK-1) belongs to the MAPKs cascade [13]. Moreover, the MAPKs cascade can be also activated through the TRIF pathway, and finally regulates the Interferon Regulatory Factor 3 (IRF-3). The MAPKs cascade regulates the post-translation of JunD gene.

Both MyD88 and TRIF signaling pathways are under the control of negative regulators such as the TNF Receptor-associated Factor 3 (TRAF-3). The latter regulates the Interferon Regulatory Factor 5 (IRF-5) which is needed for the proinflammatory cytokines genes expression. Thus, activation of the innate immune system results in the production of cytokines such as interleukins (IL) including IL-1α, IL-10. An induction of proinflammatory cytokines can be triggered through the Nuclear Factor kappa B pathway by the activation of Conserved Helix-Loop-Helix-Ubiquitous Kinase (CHUK) or IKK-α gene. Moreover, an upregulation in the transcript levels of IL-10 can be achieved by the induction of Signal Transducer and Activator of Transcription 3 (STAT-3). Finally, Heme Oxygenase-1 (HO-1) gene has also immunomodulatory properties [14].

Given the above information, the objective of this study was to determine the effect of dietary supplementation with rumen-protected methionine or lysine, as well as with a combination of these amino acids in two different ratios, on the expression of selected key-genes (NLRs, MyD88, TRIF, MAPK-1, IRF-3, JunD, TRAF-3, IRF-5, IL-1α, IL-10, IKK-α, STAT-3 and HO-1) involved in the innate immunity of dairy sheep.

## Materials and methods

### Animals and diets

Animal handling procedures were performed in accordance with protocols approved by the Agricultural University of Athens Ethical Committee of the Faculty of Animal Sciences and complied with directive 2010/63/EC on the protection of animals used for scientific purposes. Sixty 2- to 3-year-old Chios dairy ewes (*Ovis aries*) were selected from a flock of one hundred. Fifty days postpartum, the ewes were divided into 5 homogenous subgroups (n = 12) according to their fat-corrected milk yields (2.20±0.39 Kg/day), ages and body weight (BW) (63±6 Kg). Each group was assigned to one of the following five treatments (Table 1): A: basal diet (control); B: basal diet **+**6.0 g/head rumen-protected methionine; C: basal diet + 5.0 g/head rumen-protected lysine; D: basal diet **+**6.0 g/head rumen-protected methionine + 5.0 g/head rumen-protected lysine; and E: basal diet **+**12.0 g/head rumen-protected methionine + 5.0 g/head rumen-protected lysine. Both The basal diet consisted of approximately 1.1 Kg of alfalfa hay, 0.6 Kg of wheat straw and 1.5 Kg of concentrate and met the average nutritional requirements of each dietary group. The concentrate consisted of (in g/Kg): maize grain, 410; wheat middlings, 330; soybean meal, 130; sunflower, 100; mineral and vitamin premix, 30. The diet was formulated by NDS Professional software (Ver. 3.9.7.11, Rumen Sas, Reggio Emilia, Italy) by using the model for small ruminants [15]. All the diets were fed on a group basis as is traditionally done in practice. Forage was provided with the concentrate in three equal portions after milking. The sheep were milked three times per day (at 6, 13 and 21 hr) into a 12-pit milking parlor (DeLaval Corporation, Sweden). The whole experimental period lasted 75 days. After the experimental period the animals remained in the commercial farm.

For small ruminants, the amino acids requirements have not yet been determined, therefore the supplementation levels of both methionine and lysine in ewes’ diets were adjusted (metabolic BW ^0.75^), based on what has been established for dairy cows [16]. More specifically, the NRC [16] suggests that lysine and methionine percentages should be 7.2 and 2.4% of the metabolizable protein (MP) respectively (i.e., 3:1 ratio) for optimal milk protein synthesis. Furthermore, it has been indicated that, in addition to the percentages of lysine and methionine, the ratio of these two amino acids in MP is also important. For instance, increasing the percentage of lysine in MP decreases milk fat when the methionine supply is deficient or when the lysine: methionine ratio is greater than 3:1 [17]. More recently, using the breakpoint analysis, other authors showed that to maximize milk protein yield, the calculated estimates for Lys and Met (% of MP) are 7.00 and 2.60% of MP, respectively, and to maximize milk protein content, the same values are 6.77 and 2.85 (% of MP), respectively. The proper ratio between Lys and Met seems therefore to be updated to 2.69 [18]. However, the recommended percentages and ratio are typically hard to achieve in most dairy diets. The only efficient way to achieve the above recommendation is by using rumen-protected amino acids because dietary protein and crystalline amino acids are largely degraded by ruminal microbes. In this study, we used commercial products supplying rumen protected methionine and lysine which contained 60% of isopropyl ester of hydroxyl analogue of methionine (HMBi) and 68% L-Lysine monohydrochloride respectively. Thus, the average daily intake of ewes which were fed with 6 and 5 g of the commercial products of rumen protected methionine and lysine respectively was 3.6 and 3.4 g respectively, while their respective ratios for D and E diets were 2.72 and 2.33 (Table 1).

**Table 1:** Components (% of dry matter—DM-) and estimated chemical composition (% of DM) of the diet administered to the groups of dairy sheep involved in the trial.

	Diets/Groups				
Item	A	B	C	D	E
Diet components (%DM)					
Alfa alfa hay^a^	35.36	35.3	35.31	35.24	35.17
Wheat straw^b^	19,00	18.96	18.97	18.93	18.89
Concentrate mix^c^	45.63	45.54	45.55	45.46	45.37
Rumen protected methionine commercial product	-	0.2	-	0.2	0.4
Rumen protected lysine commercial product	-	-	0.17	0.17	0.17
Diet chemical composition (%DM)^d^					
DM	90.39	90.4	90.40	90.41	90.41
Ash	6.85	6.91	6.86	6.92	6.99
aNDFom	38.01	37.93	37.94	37.86	37.86
ADF	26.29	26.24	26.24	26.20	26.14
ADL	5.61	5.60	5.60	5.59	5.59
NFC	39.69	39.64	39.63	39.57	39.57
Starch	23.03	23.01	23.00	22.98	22.98
Sugars	3.22	3.22	3.22	3.21	3.21
EE	1.67	1.66	1.75	1.75	1.74
CP	13.92	13.99	13.97	14.03	14.03
Sol CP	3.69	3.78	3.69	3.78	3.78
RDP3x	8.92	8.90	8.94	8.92	8.92
Met %, (g/d)	0.23 (6.5)	0.35 (7.8)	0.23 (6.5)	0.35 (7.8)	0.35 (9.2)
Met, % MP	2.18	2.60	2.17	2.59	3.04
Lys %, (g/d)	0.60 (20.3)	0.60 (20.3)	0.72 (21.3)	0.72 (21.3)	0.72 (21.3)
Lys, % MP	6.81	6.77	7.12	7.08	7.05
Lys:Met	3.13:1	2.60:1	3.28:1	2.72:1	2.33:1
ENl (Mcal/kg)	1.53	1.53	1.53	1.53	1.53

^a^ 93.0%DM, 7.1% Ash, 14.6%CP, 10.0%EE, 51% NDF

^b^ 91.6%DM, 6.8% Ash, 16.0%CP, 8.0%EE, 72.9% NDF

^c^ 87.7%DM, 6.8% Ash, 15.30%CP, 2.8%EE, % NDF

^d^ Estimated using the software NDS Professional Ver. 3.9.7.11, Rumen Sas, Reggio Emilia, Italy.

aNDFom: Ash-free NDF treated with amylase; ADF: Acid Detergent Fiber; ADL: lignin; NFC: Non Fibrous Carbohydrate; EE: Ether Extract; CP: Crude Protein; Sol CP: Soluble Crude Protein; RDP3x: Rumen Degradable Protein at 3 times the maintenance intake; Met %, (g/d): methionine level, expressed both as % DM or daily amount; Met, % MP: methionine expressed as % of metabolizable protein; Lys %, (g/d): lysine level, expressed both as % DM or daily amount; Lys, % MP: lysine expressed as % of metabolizable protein; ENl (Mcal/kg): Net Energy of lactation, expressed as Mcal/kg.

### Blood samples

#### Blood sample collection for macrophage and neutrophil isolation

Individual blood samples were taken from the jugular vein of each sheep (10ml /animal) in each dietary treatment group (n = 60 in total) in the morning, before feeding. The samples were collected in tubes containing 17 Units/ml heparin. Specifically, two individual blood samples were randomly collected from each dietary treatment during the last 6 days of the experimental period (75 days in total) and were immediately transported into ice to the Laboratory of Nutritional Physiology and Feeding at the Agricultural University of Athens for further analysis. This same procedure was followed for several days until samples had been taken from all of the animals. Given that the commercial farm was far away from the university, that the cells (macrophages and neutrophils) should be isolated immediately (without any storage) from whole blood by using a long-term assay and that a maximum of only 10 samples could be handled each day, this sampling procedure was done only once (at the end of the experimental period).

#### Cell isolation

Monocytes and neutrophils were isolated from 60 individual blood samples as described by [10]. RNA extraction, removal of gDNA and cDNA synthesis was also performed according to [10].

#### Primers

A pair of primers specific for each target gene was designed using Geneious software (Biomatters Ltd, New Zealand) according to the respective *Ovis aries* gene coding sequences (CDS in GenBank) (S1 Table). The specificity of each pair of primers was tested against genomic DNA (positive control) to confirm that a single amplicon of 70–106 bp (S1 Table) would emerge after quantitative real-time PCR. In addition, dissociation curves were generated, and the amplification products were subjected to agarose gel electrophoresis to confirm the production of a single amplicon per reaction.

#### Real-time quantitative PCR

The relative mRNA expression levels for the target genes were quantified with a StepOnePlus^™^ Real-Time PCR System (Applied Biosystems, Foster City, CA, USA) using SYBR Select Master Mix (Applied Biosystems, Austin, TX, USA), gene-specific primers at a final concentration of 0.2 μM each (forward and reverse) and 1 μl of each cDNA as template. PCR cycling started at 95°C for 15 min followed by 40 cycles at 95°C for 15 s and 60°C for 1 min. Primer specificity and the formation of primer dimers were monitored by melt curve analysis. GAPDH and YWHAZ were used as housekeeping genes to normalize the cDNA template concentrations. The choice of the housekeeping genes was based on a study by [19] that nominated these genes as the most stable in sheep phagocytic cells. The relative expression levels of the genes of interest were calculated as (1+E)^−ΔCt^, where ΔCt is the difference between the geometric mean of the two housekeeping genes’ Cts and the Ct of the target gene, and the primer efficiency is the mean of each amplicon’s efficiency per primer, which was calculated by employing the linear regression method on the log (fluorescence) per cycle number (ΔRn) using the LinRegPCR software [20].

### Statistical analysis

Experimental data were analyzed using the SPSS statistical package (version 20.0) and are presented as means and pooled standard errors (SEM). Dietary treatment effects on the mRNA expression of genes involved in the immune system (in both monocytes and neutrophils) were explored using one-way analysis of variance (ANOVA) followed by Tukey’s multiple range test. Pearson’s correlation coefficients were used to determine the relationships between gene expression in both monocytes and neutrophils using heatmap chart. For all tests, the significance was set at 0.05. Graphs were drawn using GraphPad Prism (version 7.0), and the error bars represent the SEM.

## Results and discussion

**Fig 1:**
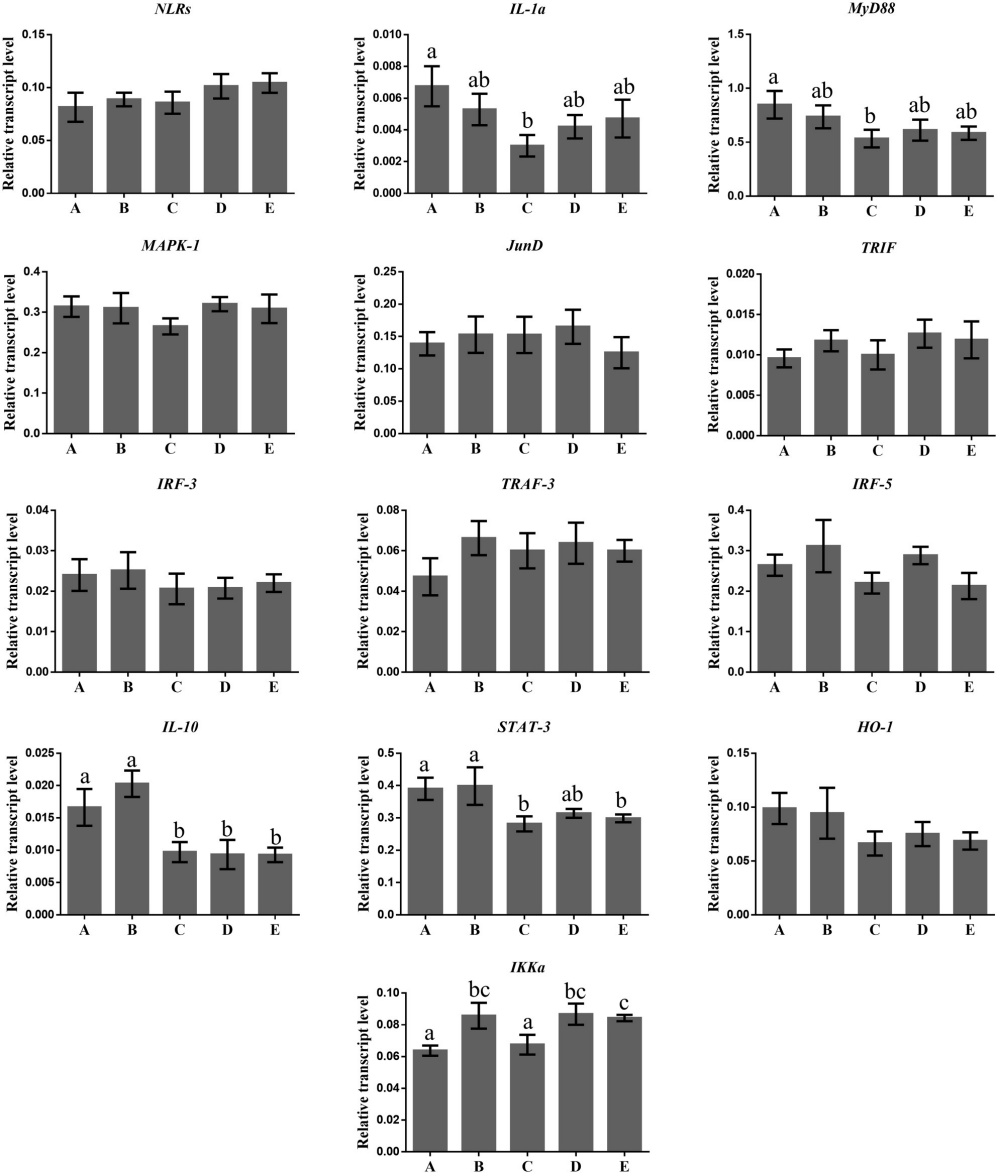
Transcript abundance of several genes in the neutrophils of sheep. NOD-like receptors (NLRs), Interleukin 1a (IL-1a), Myeloid-Differentiation-primary response gene 88 (MyD88), Mitogen-Activated Protein Kinase-1 (MAPK-1), Transcription factor JunD (*JunD*), TIR (Toll/Interleukin-1 Receptor) domain-containing adaptor protein inducing interferon beta (TRIF), Interferon Regulatory Factor 3 (IRF-3), TNF Receptor-associated Factor 3 (TRAF-3), Interferon Regulatory Factor 5 (IRF-5), Interleukin 10 (IL-10), Signal Transducer and Activator of Transcription 3 (STAT-3), Heme Oxygenase-1 (HO-1) and Conserved Helix-Loop-Helix-Ubiquitous Kinase (CHUK) or IKK-α relative to the geometrical mean of the references genes (Glyceraldehyde 3-Phosphate Dehydrogenase (*GAPDH*) and Tyrosine 3-monoxygenase/tryptophan 5-monooxygenase activation protein, zeta polypeptide (*YWHAZ*)). Bars show means ± SEM of each (n = 12) of the five dietary treatments; A: control, basal diet; B: basal diet +6.0 g/head rumen- protected methionine; C: basal diet +5.0 g/head rumen- protected lysine; D: basal diet +6.0 g/head rumen- protected methionine +5.0 g/head rumen- protected lysine and E: basal diet +12.0 g/head rumen- protected methionine +5.0 g/head rumen- protected lysine fed in ewes. Superscripts with small letters (a, b, c) between the five dietary treatments (A, B, C, D, E) differ significantly (p ≤ 0.05). The units in diagrams are relative.

**Fig 2:**
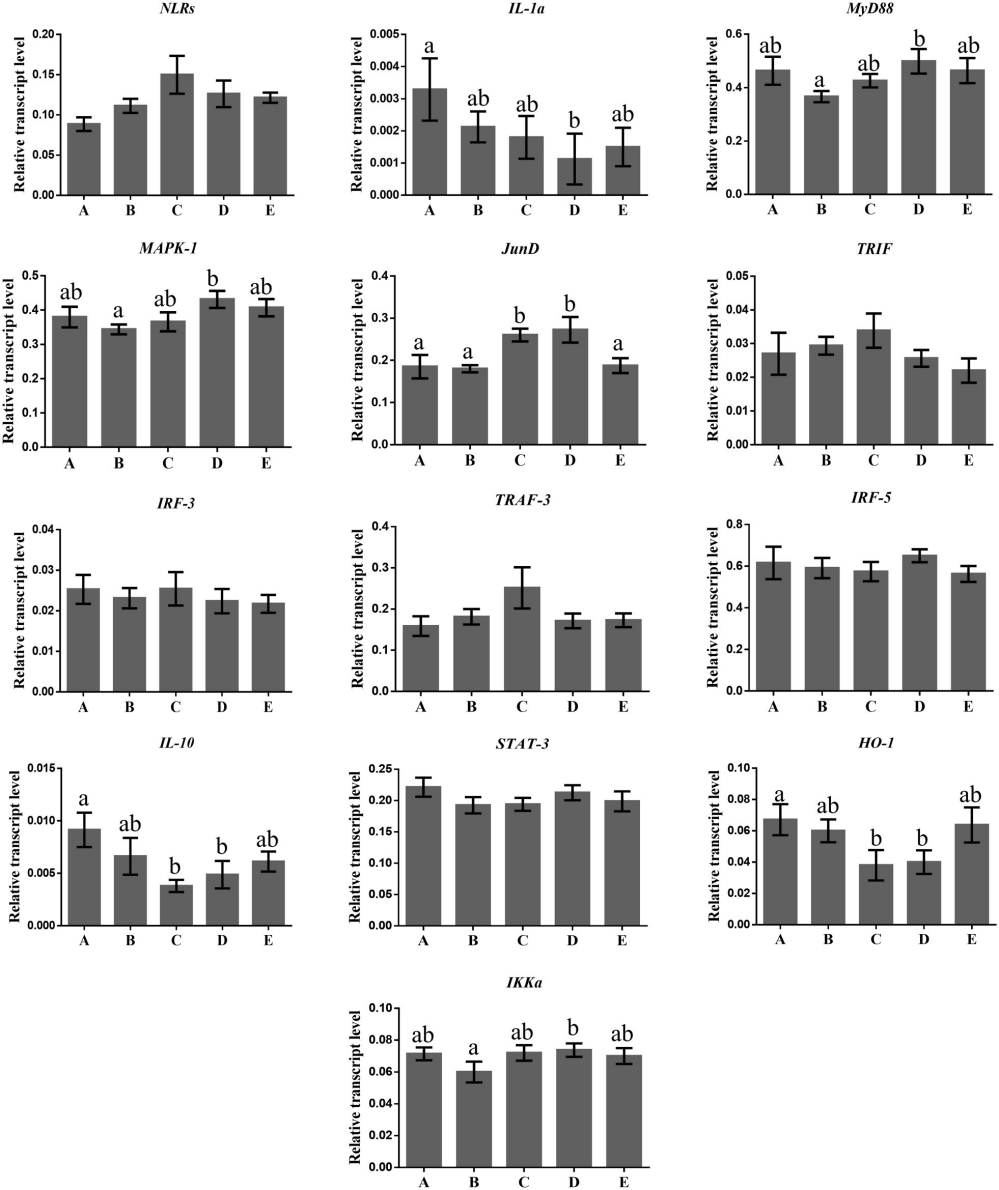
Transcript abundance of several genes in monocytes of sheep. NOD-like receptors (NLRs), Interleukin 1a (IL-1a), Myeloid-Differentiation-primary response gene 88 (MyD88), Mitogen-Activated Protein Kinase-1 (MAPK-1), Transcription factor JunD (*JunD*), TIR (Toll/Interleukin-1 Receptor) domain-containing adaptor protein inducing interferon beta (TRIF), Interferon Regulatory Factor 3 (IRF-3), TNF Receptor-associated Factor 3 (TRAF-3), Interferon Regulatory Factor 5 (IRF-5), Interleukin 10 (IL-10), Signal Transducer and Activator of Transcription 3 (STAT-3), Heme Oxygenase-1 (HO-1) and Conserved Helix-Loop-Helix-Ubiquitous Kinase (CHUK) or IKK-α relative to the geometrical mean of the references genes (Glyceraldehyde 3-Phosphate Dehydrogenase (*GAPDH*) and Tyrosine 3-monoxygenase/tryptophan 5-monooxygenase activation protein, zeta polypeptide (*YWHAZ*)). Bars show means ± SEM of each (n = 12) of the five dietary treatments; A: control, basal diet; B: basal diet +6.0 g/head rumen- protected methionine; C: basal diet +5.0 g/head rumen- protected lysine; D: basal diet +6.0 g/head rumen- protected methionine +5.0 g/head rumen- protected lysine; and E: basal diet +12.0 g/head rumen- protected methionine +5.0 g/head rumen- protected lysine fed in ewes. Superscripts with small letters (a, b, c) between the five dietary treatments (A, B, C, D, E) differ significantly (p ≤ 0.05). The units in diagrams are relative.

**Fig 3:**
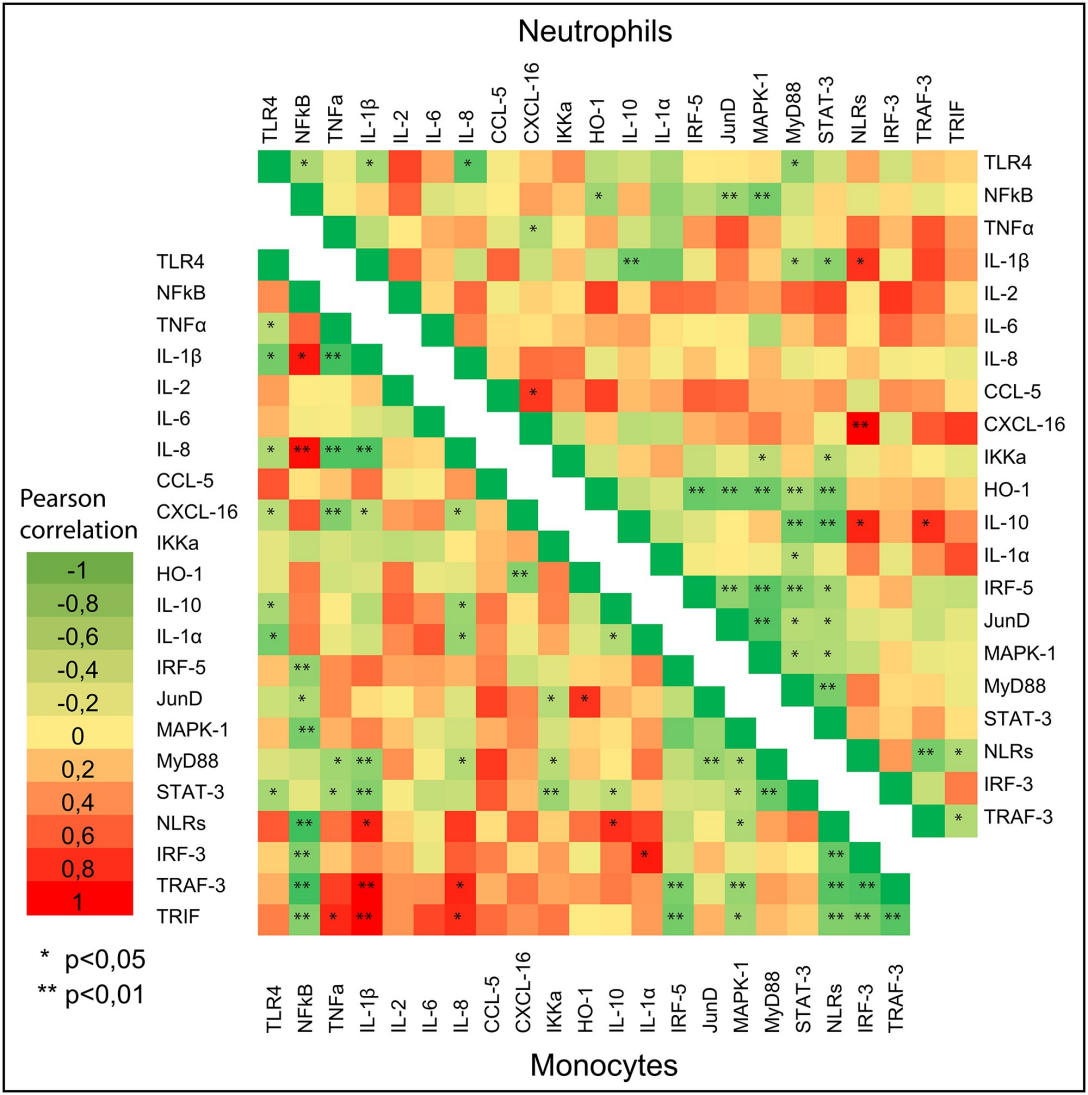
Pearson’s heat map correlations between the mRNA expression of several genes in neutrophils and monocytes of sheep. Toll-like receptors 4 (TLR-4), nuclear factor kappa B (NF-κB), tumor necrosis factor a (TNFa), interleukin 1β (IL-1β), interleukin 2 (IL-2), interleukin 6 (IL-6), interleukin 8 (IL-8), C-C motif chemokine ligand 5 (CCL-5) and chemokine (C-X-C motif) ligand 16 (CXCL-16), NOD-like receptors (NLRs), Interleukin 1a (IL-1a), Myeloid-Differentiation-primary response gene 88 (MyD88), Mitogen-Activated Protein Kinase-1 (MAPK-1), Transcription factor JunD (*JunD*), TIR (Toll/Interleukin-1 Receptor) domain-containing adaptor protein inducing interferon beta (TRIF), Interferon Regulatory Factor 3 (IRF-3), TNF Receptor-associated Factor 3 (TRAF-3), Interferon Regulatory Factor 5 (IRF-5), Interleukin 10 (IL-10), Signal Transducer and Activator of Transcription 3 (STAT-3), Heme Oxygenase-1 (HO-1) and Conserved Helix-Loop-Helix-Ubiquitous Kinase (CHUK) or IKK-α relative to the geometrical mean of the references genes (Glyceraldehyde 3-Phosphate Dehydrogenase (*GAPDH*) and Tyrosine 3-monoxygenase/tryptophan 5-monooxygenase activation protein, zeta polypeptide (*YWHAZ*)).

In this study, no significant reduction in the mRNA expression levels of NLRs gene, in the neutrophils of ewes fed with rumen protected amino acids compared with the control ones, was found (Fig 1). This reduction was accompanied by a decline in the mRNA transcript accumulation levels of IL-1α gene which was significant in the case of ewes fed with the lysine in comparison with the controls (Fig 1). A down regulation of IL-1α gene in the monocytes of treated ewes was also observed, but the results were significant only for those fed simultaneously with methionine and lysine at a ratio 6 to 5 g/day /animal (Fig 2). Both macrophages and neutrophils express several families of pathogens recognition receptors (PRRs) such as NLR [21] which are involved in the formation and activation of inflammasomes, a signaling pathway mediating the secretion of IL-1α [12, 22]. Oxidative stress [23], lipid overload [24], and microbial infections, which activate the Toll-like receptors (TLR) family, [25] are also factors that induce IL-1α gene expression [26]. The NF-κ B transcription factor can also upregulate the IL-1α gene expression [27]. Thus, the significant reduction in the mRNA transcripts accumulation levels of TLR-4 gene in the neutrophils of treated ewes, which has previously been reported [10], verify the close relationship among TLR-4, NF-κB and IL-1α. This relationship was also confirmed, by the significantly positive correlations, which were found between the expression levels of TLR-4 and NF-κB genes in the neutrophils and TLR-4 and IL-1α genes in monocytes (Fig 3). Moreover, the inhibitory effects of lysine alone or in combination with methionine (at a ratio 5/6) in the neutrophils and monocytes respectively, on the mRNA expression levels of IL-1α gene, support the assumption of their anti-inflammatory role since the IL-1α has pro-inflammatory properties.

Signal transduction by PRRs depends on their conjunction with specific adaptors [12]. MyD88 is a common adaptor for all the TLRs, except for TLR-3 [28]. Lower mRNA relative expression levels of MyD88 gene in the neutrophils of ewes treated with rumen protected amino acids, in comparison with the normally fed, were found but the results were statistically significant for those consumed the lysine only (Fig 1). These findings are in accordance with the significantly lower transcripts accumulation levels of TLR-4 gene in the neutrophils of ewes fed with the same dietary treatments, as previously reported [10]. Indeed, a significantly positive correlation was found in the neutrophils of ewes between MyD88 and TLR-4 genes (Fig 3). Thus, it seems that the positive feedback loop of TLR-4- dependent molecular self-regulation, of the downstream signaling of MyD88, could at least partly explain our data. A significant enhancement in the mRNA relative expression levels of TLR-4 and MyD88 genes has been observed in goats’ mammary epithelial cells [29], and in human endometrial cells [30] when stimulated *in vitro* by gram- negative lipopolysaccharide bacteria cell wall components. MyD88 show also highly induced transcription levels in bovine mastitis tissue [31]. Thus, our results concerning MyD88 gene, may further support the idea of the absence of any inflammation in clinical or subclinical form especially for the case of lysine fed ewes.

Activation of MyD88-depended pathway might affect the MAPKs cascade [13]. Indeed, the expression levels of MyD88 and MAPK-1 gene were positive correlated in both ewes’ neutrophils and monocytes (P<0.05) (Fig 3). In this study, the mRNA relative expression levels of MAPK-1 gene, in both neutrophils (Fig 1) and monocytes (Fig 2), were not affected significantly between the treated and the control ewes. However, it should be pointed out here that the dietary supplementation level with methionine only, resulted in significantly lower mRNA accumulation relative levels of MAPK-1 gene in the monocytes of ewes, in comparison with those fed simultaneously with methionine and lysine at a ratio of 6/5 g/head/day (Fig 2). Pathogens, stress or endogenous inflammatory factors released from necrotic cells bind to TLRs and NLRs which stimulate the MAPK cascade through the MyD88 pathway [32], and trigger the cytokines production [33, 34]. Thus, the unaffected mRNA expression level of MAPK-1 gene together with the downregulation of the mRNA expression levels, of both NLRs and IL-1α in the neutrophils of treated ewes, could as well highlight an improvement of their innate immune system.

A significant enhancement in the transcript accumulation level of JunD gene in monocytes of ewes fed either with lysine alone or with a combination of methionine with lysine at a ratio 6/5 g/ewe/day, compared with the control ones was found (Fig 2). The MAPKs cascade regulates the post-translation of activating protein-1 (AP-1) which its major part is the JunD subfamily. Notwithstanding that the dietary supplementation with rumen protected amino acids did not affect mRNA expression level of MAPK-1 gene, as mentioned earlier evidence confirms the involvement of JunD gene in the IL-1β synthesis in primary macrophages of mouse [35]. Actually, a significant downregulation in the mRNA accumulation level of IL-1β genes has previously been found in both monocytes and neutrophils of treated ewes [10], which may underline the negative regulator role of JunD gene in IL-1β signaling pathway. JunD gene plays also an important role in the defense against oxidative stress [36]. It has been indicated that inactivation of JunD gene causes chronic oxidative stress [37, 38]. Moreover, the nonappearance of JunD gene in mice resulted into upregulation of hypoxia inducible factor genes in their kidney [39]. Additionally, the over expression of JunD gene not only reduces ROS production, H_2_O_2_, and hypoxia inducible factor (HIF) protein level but also the mRNA expression level of VEGF-A gene [40]. The fact that these amino acids are involved in key antioxidant mechanisms [41], may explain our results on the expression of the JunD gene.

Although MyD88 is a common adaptor for all the TLRs except TLR-3, TRIF is an adaptor for TLR-3 and TLR-4, which promotes an alternative pathway that leads to the activation of IRF3, NF-κB and MAPKs for induction of type IFN and inflammatory cytokines [42, 43]. However, in this study, no differences were found on the mRNA expression levels of TRIF gene in both neutrophils (Fig 1) and monocytes (Fig 2) of ewes among the dietary treatments, although a downregulation on TLR-4 and NF-κB genes, have previously been reported in these cells types [10]. Additionally, the dietary supplementation with rumen protected amino acids had no effect, in the mRNA transcripts accumulation level of IRF-3 gene in both ewes’ neutrophils (Fig 1) and monocytes (Fig 2). Higher mRNA expression levels of IRF-3 gene were found in goats mammary epithelia cells, when incubated *in vitro* for 3 h with both toxins from Gram-negative lipopolysaccharide and Gram-positive lipoteichoic acid bacterial [29]. The same trend has been found in bovine mammary epithelial cells when stimulated either with *Escherichia coli* or *Staphylococcus aureus* [44]. Moreover, the pro-inflammatory role of IRF-3 has been confirmed also in mice macrophages, through the activation of TLR-4–TRIF metabolic pathway which induces the production of pro-inflammatory cytokines [45]. Thus, the unaffected expression level of IRF-3 gene, in this study, may be due to the nonappearance of any inflammatory incident. Moreover, these results concerning the expression of TRIF and IRF-3 genes may indicate that the MyD88-independent pathway was not affected by the dietary inclusion of rumen protected amino acids.

Both the MyD88 and TRIF pathways are controlled by TRAF regulators such as TRAF-3 [46, 47]. A negative relationship exists between TRAF-3 regulator with the pro-inflammatory cytokines, which their production is regulated by the TLR pathway [12]. Interestingly, it has been observed, that genetic deficiency of TRAF, promotes inflammation incident in mice [48, 49]. In our study, numerically higher mRNA expression levels of TRAF-3, in both neutrophils (Fig 1) and monocytes (Fig 2) of ewes, supplemented with rumen protected amino acids, compared with the control one were found. The mRNA accumulation levels of both TRAF-3 and IL-1α genes, together with those which have previously been reported for the TLR-4 gene [10] validate the negative regulating role of TRAF-3 to the TLR-stimulated expression of pro-inflammatory cytokines pathway.

The TLR-4—MyD88 dependent signaling pathway can also regulate the IRF-5 gene expression [50]. IRF-5 gene regulates inflammation and immune responses and it is mainly expressed in macrophages and monocytes cells [51]. In this study, the mRNA transcripts accumulation levels of IRF-5 gene were not affected by the dietary inclusion of rumen protected amino acids in both neutrophils (Fig 1) and monocytes (Fig 2) of ewes, although a downregulation in the relative transcripts of TLR-4 gene has previously been observed in these cells [10]. A significant induction in the mRNA relative expression levels of IRF-5 gene has been observed in hematopoietic cells of mice [50] and human and/or bovine kidney cells [52] *in vitro*, when stimulated by gram- negative lipopolysaccharide bacteria cell wall components. Thus, the IRF-5 gene expression may strengthen further the idea of absence of any inflammation in clinical and/or subclinical form.

In this study, a significant downregulation of IL-10 gene was found, in the neutrophils of ewes fed either with lysine alone or combined with methionine at ratios 5/6 and 5/12 g/head/ewe respectively, in comparison with the control (Fig 1). Significant was also the downregulation of IL-10 gene in the monocytes of ewes fed either with lysine alone or simultaneously with methionine, at a ratio of 5/6/day /ewe compared with the control ones (Fig 2). PRRs, stress and endogenous inflammatory factors released from necrotic cells upregulate the mRNA expression level of IL-10 gene in the innate immune cells [28]. Indeed, significantly higher mRNA expression level of IL-10 gene has been found in bovine mastitis tissue [31] while, in mouse models the over expressed IL-10 gene is related with immunosuppressive incidents [53]. It has been shown that upregulation of mRNA transcripts levels of IL-10 gene in macrophages is mediated by the recruitment of STAT-3 into the IL-10 promoter [54, 55]. In this study, significantly lower were, also, mRNA accumulation levels of STAT-3 gene in the neutrophils of ewes fed with either lysine only or with a combination of methionine with lysine at a ratio of 12/5 g/day/ewe in comparison with the control ones (Fig 1). Additionally, a significantly positive correlation between the mRNA expression levels of IL-10 and STAT-3 genes were found (Fig 3). Thus, the results as the IL-10 and STAT-3 genes expression is concerned may not only support their close relationship through the IL-10/Janus kinase (JAK)/STAT-3 pathway, but also their negative regulation in both acute and chronic inflammation.

HO-1 gene has also been recognized for its immunomodulatory properties [14]. The enzyme which is produced from this gene catalyzes the reaction that produces biliverdin, ferritin and carbon monoxide from free heme which is toxic for the cells and promotes inflammation [56]. Lower mRNA transcripts accumulation level of HO-1 gene has been observed in both neutrophils (Fig 1) and monocytes (Fig 2) of treated ewes but the results were significant for the monocytes of ewes fed either with lysine only, or simultaneously with methionine and lysine at ratio 6/5 compared with the controls (Fig 2). A significant upregulation in the transcript levels of HO-1 gene has been found in mice macrophages, after their stimulation with Gram-negative lipopolysaccharide bacteria. Significantly higher mRNA expression levels have also been observed in the liver of bovine and mice, infected by *Fachiola hepatica* [57]. STAT-3 seems to be involved in the metabolic pathway by which IL-10 induces the HO-1 gene expression [58]. Moreover, TLR-4 and NF-κB pathways are also involved in the regulation of HO-1 gene [14]. Thus, referring to the results on the mRNA expression levels of TLR-4, NF-κB, STAT-3, IL-10 and HO-1 genes of this study, an enhancement of the innate immune responses by the dietary supplementation with rumen protected amino acids could be claimed.

A significant enhancement in the mRNA transcript accumulation level of IKK-α (CHUK) gene in the neutrophils of ewes fed either with methionine only, or simultaneously with methionine and lysine at ratios of 6/5 and 12/5 g/ewe/day respectively compared with the control ones (Fig 1), was observed. Moreover, significant higher mRNA expression level of IKK-α gene was found in the monocytes of ewes supplemented simultaneously with methionine and lysine at a ratio of 6/5 g/ewe/day in comparison with those fed with methionine only (Fig 2). IKK-α regulates the “alternative” NF-κB pathway [59], although the role of this pathway in the inflammation is still unclear [59, 60]. Moreover, the activation of NF-κB through the “canonical” pathway is negatively regulated by the IKK-α, as it has been indicated in macrophages derived from fetal liver cells of IKK-α knockout embryo [61]. It has been found, also, in mice that IKK-α limits macrophages NF-κB activation and contributes to the resolution of inflammation [62]. More specifically, IKK-α contributes to suppress NF-κB by accelerating both the turnover of the NF-κB subunits ReIA and c-Rel, and their removal from inflammatory gene promoter. Increased expressions of pro-inflammatory cytokines have been found in IKK-α deficient macrophages [61], while inactivation of IKK-α gene enhances inflammation in mice [62]. IKK-α has anti-inflammatory role through the regulation of SUMO ligase activity of protein inhibitor of activated STAT1 (PIAS) [63]. IKK-α through the phosphorylation of PIAS blocks the connection of both STAT1 and NF-κB to pro- inflammatory genes promoters [63]. However, it should be pointed out here that the dietary supplementation with rumen protected amino acids had no effect on the mRNA transcript accumulation level of NF-κB gene in both neutrophils and monocytes of ewes as previously has been indicated [10]. Thus, the results concerning the mRNA expression levels of both IKK-α and NF-κB genes, support further the assumption of stronger innate immunity in ewes when supplemented with rumen protected amino acids.

## Conclusions

The significant downregulation in the transcripts accumulation levels of genes, which their high expression is usually related with inflammation incidents, in the neutrophils (IL-1α, IL-10, MyD88 and STAT-3) and in monocytes (IL-1α, IL-10 and HO-1) of ewes fed daily with 3.4 g lysine alone, or with a combination of 3.6 g methionine and 3.4 g lysine, compared with the control, may indicates its anti-inflammatory role. Moreover, the significant induction in the relative transcript levels genes (JunD and IKK-α) with anti-inflammatory role in the monocytes of ewes fed with methionine and lysine at a ratio 6/5 compare with the control may further support the hypothesis of the absence of any inflammation in clinical and/or subclinical form. Finally, the rumen protected amino acids were not affecting the MyD88 independent pathway in both monocytes and neutrophils of ewes.

## Supporting information

S1 Table Sequences and amplicon size of primers used in real-time qPCR. Primer were designed as previously described in “Material and methods” for the purpose of the present study.
(DOCX)
