# Supplementary material for: The impact of rumen-protected amino acids on the expression of key- genes involved in the innate immunity of dairy sheep
Source: PLoS One. 2020 May 14;15(5):e0233192. doi: 10.1371/journal.pone.0233192 (PMC7224535; doi:10.1371/journal.pone.0233192)
Supplement: S1 Table — Primer were designed as previously described in “Material and methods” for the purpose of the present study. (DOCX) [file pone.0233192.s001.docx]

S1 Table. Sequences and amplicon size of primers used in real-time qPCR.

| Gene | Sequence | Amplicon bp |
| --- | --- | --- |
| *GAPDH* | F: 5'-AAAGCCATCACCATCTTCCA -3' | 74 |
|  | R: 5'-ACCACGTACTCAGCACCTCAT-3' |  |
| *YWHAZ* | F: 5'-TGTTCTATTGTGCCTAGTACACTGT -3' | 70 |
|  | R: 5'-CATCAAGACTCACTGCCTCCC -3' |  |
| *NLRs* | F: 5'- CAACCTACTCCACGACCAGG -3' | 96 |
|  | R: 5'- TGGATGAAGTTCCACTGCA -3' |  |
| *IL-1a* | F: 5'- TCAAGCCCAGATCAGCACAT -3' | 100 |
|  | R: 5'- TGATTGAGGGCGTCGTTCAG -3' |  |
| *MyD88* | F: 5'- ACAGACAAACTATCGGCTGA-3' | 99 |
|  | R: 5'- CACCTCTTCTCAATGAGTTCA-3' |  |
| *MAPK-1* | F: 5'-GCAACGACCACATCTGCTAC-3' | 100 |
|  | R: 5'-AGGTTGGAAGGCTTGAGGTC-3' |  |
| *JunD* | F: 5'-ACGCAGTTCCTCTTTCCCAA-3' | 100 |
|  | R: 5'-CCAGCTGGTTTTGCTTGTGT-3' |  |
| *TRIF* | F: 5'- GCACGTCTAGCCTGCTTAC -3' | 106 |
|  | R: 5'- TTGCGGGCCCGCAGCATCT -3' |  |
| *IRF-3* | F: 5’-CCAGAGGCTGGGGCACTGCC-3’ | 92 |
|  | R:5’-CCTTCGGGACCTCGCCGTCA-3’ |  |
| *TRAF-3* | F: 5'- TAACTGCTGCATTCGCTCCA -3' | 100 |
|  | R: 5'- GGAACACAAAGCTGGGGTTG -3' |  |
| *IRF-5* | F: 5'-ACATCCCCAGTGAGAAGCAG-3' | 100 |
|  | R:5'-ATGGCATACAGATCCTGGCC-3' |  |
| *IL-10* | F: 5'-CTGGGGGAGAAGCTGAAGAC-3' | 100 |
|  | R:5'-CTCTCTTCACCTGCTCCACC-3' |  |
| *STAT-3* | F: 5'-CGCAATTAGGCAGAGCAACTG-3' | 77 |
|  | R: 5'-CCCTGTATCAGAGACCATCCCA -3' |  |
| *HO-1* | F: 5'- GAGCTGACCCGAGAAGGTTT -3' | 100 |
|  | R: 5'- AGACGGGGTTCTCCTTGTTG -3' |  |
| *ΙΚΚα (CHUK)* | F: 5'-TGCAGGGAAAGAGGCAGAAA-3' | 70 |
|  | R: 5'-GACCGAGCAGAACTCTGTGT-3' |  |

Primer were designed as previously described in “Material and methods” for the purpose of the present study.
